# Silencing of δ‐aminolevulinic acid dehydratase via virus induced gene silencing promotes callose deposition in plant phloem

**DOI:** 10.1080/15592324.2021.2024733

**Published:** 2022-01-07

**Authors:** Nabil Killiny, Shelley E. Jones, Pedro Gonzalez-Blanco

**Affiliations:** Citrus Research and Education Center and Department of Plant Pathology, IFAS, University of Florida, Lake Alfred, FL, USA

**Keywords:** *Δ*‐aminolevulinic acid dehydratase, callose, phloem, citrus, virus-induced gene silencing, *citrus tristeza virus*

## Abstract

The *δ*-aminolevulinic acid dehydratase (ALAD) enzyme is an intermediate in the biosynthetic pathway of tetrapyrroles. It combines two *δ*‐aminolevulinic acid (*δ*‐ALA) molecules to form the pyrrole, porphobilinogen, an important precursor for plant pigments involved in photosynthesis, respiration, light-sensing, and nutrient uptake. Our recent efforts showed that, in citrus, silencing of *ALAD* gene via *Citrus tristeza virus-*induced gene silencing, caused yellow spots and necrosis in leaves and in developing new shoots. Silencing of *ALAD* gene reduced leaf pigments and altered leaf metabolites. Moreover, total phenolic content, H_2_O_2,_ and reactive oxygen species (ROS) increased, indicating that silencing of *ALAD* induced severe stress. Herein, we hypothesized that conditions including lower sucrose, elevated ROS, alteration of microRNA involved in RNAi regulatory protein Argonaute 1 (AGO1) and ROS lead to higher deposition of callose in phloem tissues. Using aniline blue staining and gene expression analysis of callose synthases, we showed significant deposition of callose in *ALAD*-silenced citrus.

Virus-induced gene silencing (VIGS) is a biotechnological approach that has been broadly adopted for studying plant functional genomics.^1,2^ A mild strain (T36) of the *Citrus tristeza virus* (CTV) has been developed as a VIGS vector for *Citrus spp*.^3–6^ The targeted repoter genes of citrus include those for phytoene desaturase and *δ*-aminolevulinic acid dehydratase due to their respective roles in carotenoid and chlorophyll biosynthesis and photosynthesis.^7,8^

Delta (*δ*)-aminolevulinic acid dehydratase (ALAD), a key enzyme in tetrapyrrole synthesis, joins two *δ*-aminolevulinic acid (*δ*-ALA) molecules through a condensation reaction to form the pyrrole molecule, porphobilinogen, an important intermediate for chlorophyll biosynthesis.^8^ In our previous work, we produced *ALAD*-silenced citrus using a truncated antisense *ALAD* in the binary vector pCAMBIA‐1380 containing the infectious cDNA clone of CTV isolate T36 (GenBank accession no. AY170468).^9^ The plasmid, CTV-t*ALAD*-as was propagated in *Nicotiana benthamiana* and the purified virions were infiltrated in *Citrus macrophylla*. The empty plasmid, CTV-wt was inoculated in *C. macrophylla* to be used as a control in our study.^9^ *C. macrophylla* plants inoculated with CTV-t*ALAD*-as virions showed a specific phenotype including yellow spots and necrosis in leaves, stems, and the apical meristem. For leaves, the phenotype starts as a few spots in developing young leaves and the number of spots and intensity increase with maturity, and often connect to each other until they cover most of the leaf surface.^9^ Inoculation with CTV-wt does not cause any of the described phenotype in *ALAD*-silenced plants.^9^

When the *ALAD* gene was silenced in *C. macrophylla* using CTV-t*ALAD*-as, several effects were observed: (1) *δ*-ALA accumulated, (2) the levels of chlorophylls, starch, sucrose, and pigments (*trans-* and *cis-*violaxanthin, and *α-* and *β-*cryptoxanthin) were reduced, (3) phytohormones including salicylic acid and jasmonic acid levels increased, and (4) emission of some released volatile sesquiterpenes such as *(E)-α*-bergamotene and *(E)-β-*farnesene increased.^9^ These responses indicated the gene silencing-induced stresses. In addition, polar metabolites including L-alanine, gamma-aminobutyric acid, L-threonine, L-glutamic, and L-phenylalanine also were significantly decreased in leaves while L-asparagine, fumaric acid, and succinic acid significantly increased in leaves of *ALAD*-silenced plants.^9^ Furthermore, expression profiles of 63 conserved microRNAs (miRNA) implicated in auxin biosynthesis and signaling, axillary shoot meristem formation and leaf morphology, starch metabolism, and oxidative stress were differentially expressed in *ALAD*-silenced citrus plants.^10^ Levels of total phenolics, H_2_O_2_ and superoxide anion (O_2_**^‧^**^−^) were increased, further supporting the hypothesis that *ALAD* silencing-induced stress on citrus plants.^10^

As a response to both biotic and abiotic stresses, thickening of the cells walls via callose deposition can occur in plants.^11,12^ Folimonova and Achor^13^ and Koh, Zhou, Williams, Park, Ding, Duan and Kang^14^ observed mild to excessive deposits of callose material (*β-*1,3-glucan polysaccharide) in the sieve elements of citrus plants infected with ‘*Candidatus*. Liberibacter asiaticus’, the bacterial pathogen associated with citrus greening disease. Callose deposition is thought to help restrict the movement of pathogens within the plant, and has been shown to be controlled by multiple callose synthase genes.^15,16^ In *Citrus*, nine genes for callose synthase were recently identified, and upregulation of gene expression was correlated with ‘*Ca*. L. asiaticus’ infection.^16^ In addition, callose deposition can respond to factors including light intensity, antioxidant supplementation, and sucrose concentration. Callose formation was enhanced in growth media with lower sucrose (1% compared to 5% sucrose) in *Arabidopsis*.^11^

Thus, we hypothesized that the combination of increased ROS, altered miRNAs, and reduced photosynthates including chlorophyll, carotenoids, and sucrose from the *δ*-ALA-deficient citrus model will lead directly to increased callose deposition. To test this hypothesis, we stained the phloem tissue of *C. macrophylla* inoculated with CTV-wt or CTV-t*ALAD-*as expressing varying degrees of phenotype using aniline blue.^17^ The plants used for the current study were produced for our previous study.^9^ We examined the effect of *ALAD* silencing on the gene expression of citrus callose synthases and the density of callose deposition in citrus leaves expressing a gradient of phenotypes scaled from zero (CTV-wt) and 1 to 5 (CTV-t*ALAD*-as) (Figure 1). The aniline blue staining revealed significant depositions of callose in phloem tissue when peeled barks were stained. Higher levels of callose deposition were observed in the moderate phenotype (degree 3) in *ALAD*-silenced plants while it was barely observed in control plants (Figure 1). In addition to the peeled bark, callose deposition was also observed in the phloem tissue in cross sections of the petiole in *ALAD*-silenced plants (Figure 2a–h).

**Figure 1.**
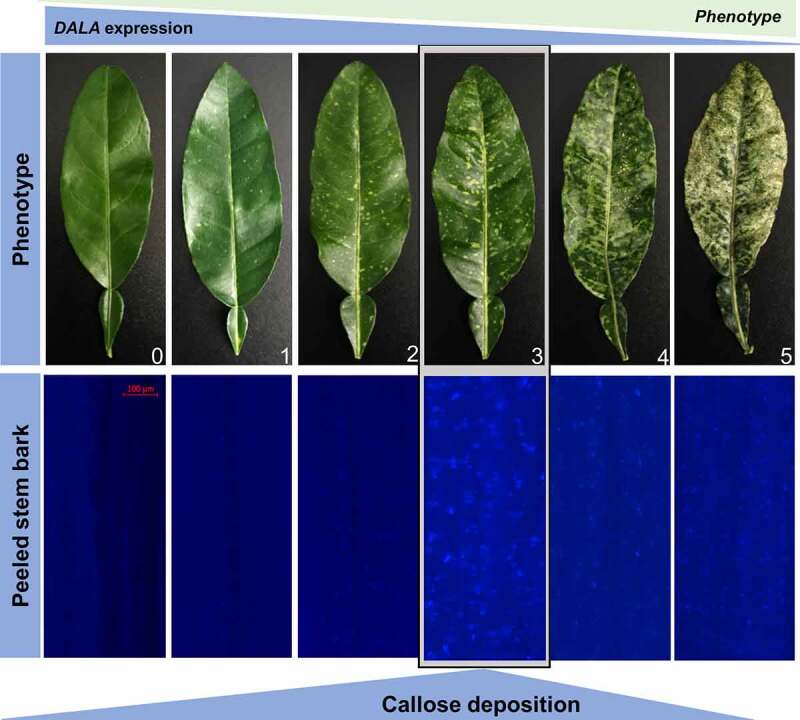
Silencing of δ‐aminolevulinic acid dehydratase via *Citrus tristeza virus-*induced gene silencing (CTV-IGS) causes callose deposition in citrus phloem tissue. Note the increased amount of callose deposits in the phloem tissue as seen in peeled stem bark is correlated with the increase in phenotype, but the highest amount is found in the moderate phenotype. Callose deposits were visualized by staining with aniline blue. Zero: control plants (CTV-wt). 1–5: degrees of phenotype in *ALAD*-silenced plants (CTV-t*ALAD*-as).

**Figure 2.**
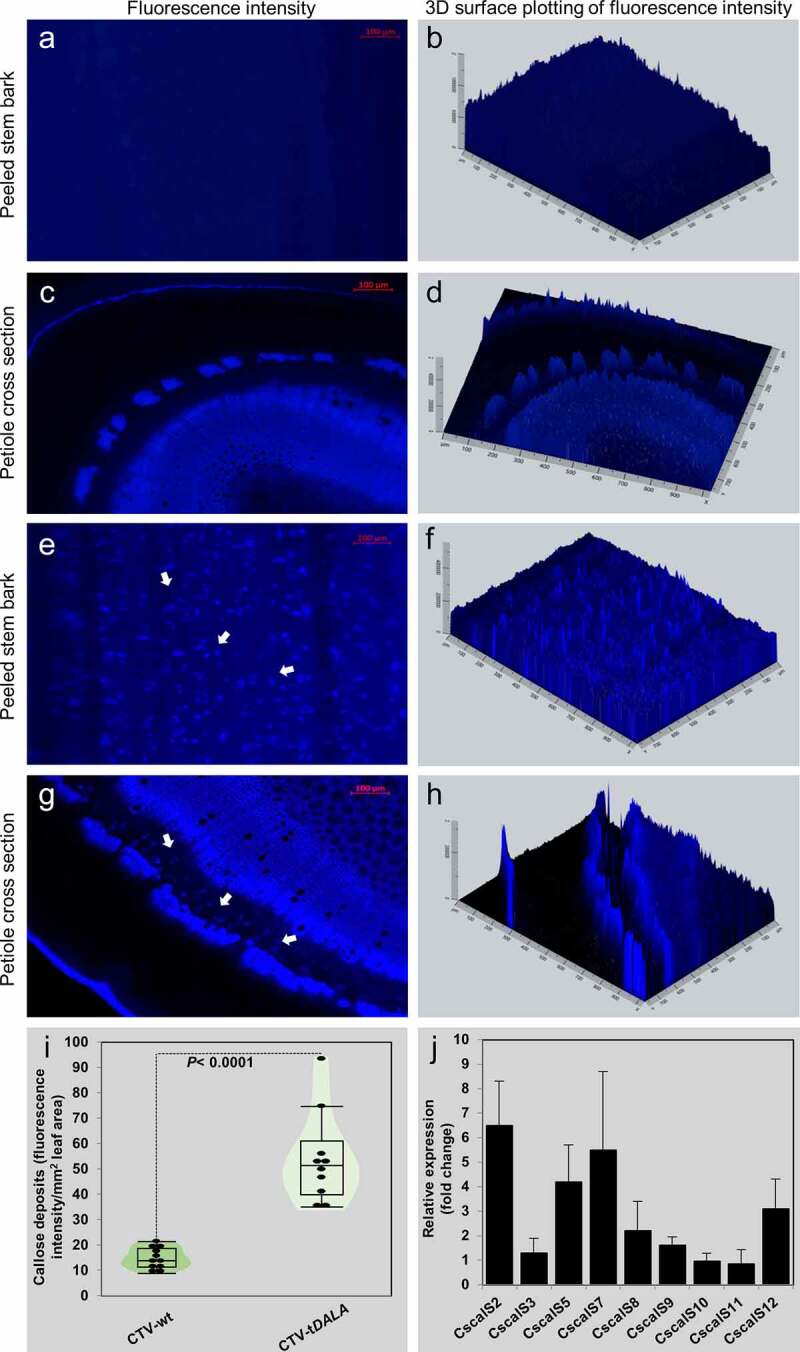
Visualization and quantification of callose deposits in the moderate phenotype of *ALAD*-silenced plants (CTV-t*ALAD*-as) phloem tissue compared to control plants (CTV-wt). A-H: Visualization of callose deposits by staining with aniline blue. A-D: Control plants. E-H: *ALAD*-silenced plants. I: Total callose deposits quantified as fluorescence intensity. J: Fold change in callose synthase gene expressions performed with RT-PCR. Arrows indicate callose deposits.

To quantify the density of callose deposition, we used ZEISS ZEN 3.1 blue edition image processing software. The total fluorescence signal density was significantly higher in *ALAD*-silenced *C. macrophylla* than the control plants (Figure 2i). Furthermore, the expression of callose synthase genes were upregulated in *ALAD*-silenced *C. macrophylla* (Figure 1j). We used the previously published primers to perform the gene expression analysis as described by Granato, Galdeano, D’Alessandre, Breton and Machado.^16^

In summary, this current work provides evidence that callose deposition occurs in *ALAD*-silenced *C. macrophylla*. By integrating the present findings and our published work,^9,10^ we illustrated a hypothetical model for callose formation in *ALAD*-silenced plants (Figure 3). In this proposed model, silencing *ALAD* causes shifts in the microRNA profile prompting the reactive oxygen species (ROS) and RNAi regulatory protein Argonaute 1 (AGO1).^10^ The last two promote callose synthesis by activation of callose synthases.^18^ On the other hand, the reduced photosynthesis caused by the downregulation of *ALAD* produces lower amounts of sucrose.^9^ Abscisic acid stimulates the callose deposition in the presence of low sucrose concentrations.^18^

**Figure 3.**
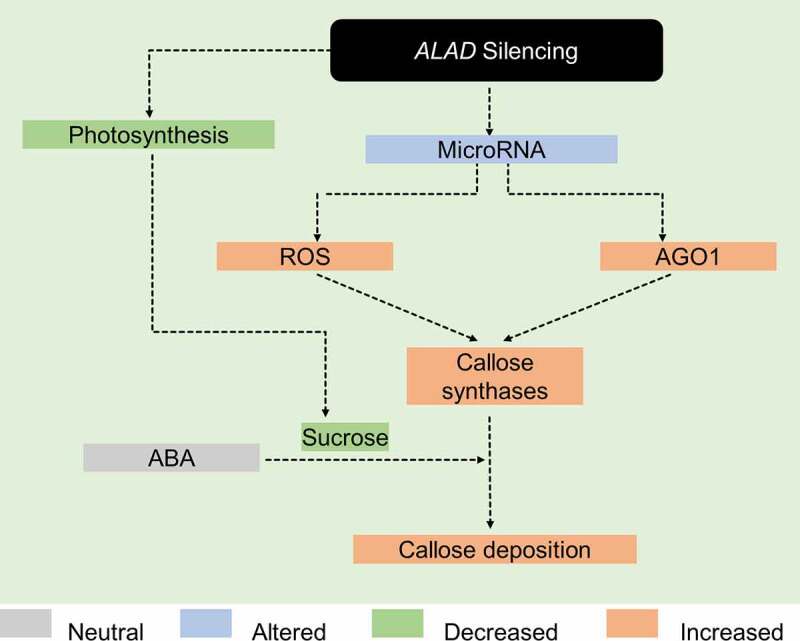
Proposed model on how silencing of δ‐aminolevulinic acid dehydratase leads to deposition of callose. See main text for details.

Finally, we assume that the callose deposition reported for ‘*Ca*. L. asiaticus’-infected citrus may not only be stimulated by the presence of the citrus greening pathogen, but that the reduction of chlorophyll pathway metabolites, increased ROS, altered miRNAs and reduced sucrose levels also contribute to callose formation. Further investigation is required to fully understand the interplay of these mechanisms and to prove the proposed hypotheses.
